# Insights into Enhanced Peroxydisulfate Activation with B and Fe Co-Doped Biochar from Bark for the Rapid Degradation of Guaiacol

**DOI:** 10.3390/molecules28227591

**Published:** 2023-11-14

**Authors:** Jian Huang, Yu Zhu, Huiyang Bian, Liang Song, Yifan Liu, Yuancai Lv, Xiaoxia Ye, Chunxiang Lin, Xiaojuan Li

**Affiliations:** 1 College of Environment & Safety Engineering, Fuzhou University, Fuzhou 350108, China; hj@fzu.edu.cn (J.H.); zy12500947@outlook.com (Y.Z.); t23087@fzu.edu.cn (L.S.); yfanym@fzu.edu.cn (Y.L.); yclv@fzu.edu.cn (Y.L.); yexiaoxia@fzu.edu.cn (X.Y.); 2 Jiangsu Co-Innovation Center of Efficient Processing and Utilization of Forest Resources, Nanjing Forestry University, Nanjing 210037, China; hybian1992@njfu.edu.cn

**Keywords:** Masson pine bark, biochar, peroxydisulfate activation, B/Fe co-doping

## Abstract

A boron and iron co-doped biochar (B-Fe/biochar) from Masson pine bark was fabricated and used to activate peroxydisulfate (PDS) for the degradation of guaiacol (GL). The roles of the dopants and the contribution of the radical and non-radical oxidations were investigated. The results showed that the doping of boron and iron significantly improved the catalytic activity of the biochar catalyst with a GL removal efficiency of 98.30% within 30 min. The degradation of the GL mainly occurred through the generation of hydroxyl radicals (·OHs) and electron transfer on the biochar surface, and a non-radical degradation pathway dominated by direct electron transfer was proposed. Recycling the B-Fe/biochar showed low metal leaching from the catalyst and satisfactory long-term stability and reusability, providing potential insights into the use of metal and non-metal co-doped biochar catalysts for PDS activation.

## 1. Introduction

The refractory organic pollutants discharged from wastewater result in great harm to the ecological environment and human health [1,2]. Therefore, many advanced treatments have been applied to reduce the adverse consequences caused by organic pollutants [3,4,5,6]. Among them, advanced oxidation processes (AOPs), especially the sulfate radical-based AOPs that involve a catalytic activation of peroxydisulfate (PDS) or peroxymonosulfate (PMS), have been considered a promising technology for the treatment of organic compounds that contain wastewater. This is because they can produce SO_4_^−^ with a high redox potential (2.5–3.1 V) under a wide pH range (2.0–11.0) [7], which is favorable for practical applications. In the process of persulfate-based AOPs, radicals including ·OH and SO_4_· can be produced by the activation of PDS or PMS assisted through some physical or chemical techniques (such as UV irradiation, heat, or catalysts) to break the O–O bond in persulfate [8]. Diverse catalysts, such as metal oxides [9], carbon materials [10], and natural minerals [11], have been exploited to catalyze persulfate. Among them, carbon materials derived from biomass waste, e.g., crop straw (which is often named biochar), show great advantages in being easily available in raw materials, having a tunable structure, and being industrially and economically attractive [12]. Nevertheless, the catalytic efficiency of pristine biochar is relatively lower than that of transition metal-based catalysts. Thus, many efforts have been made to enhance the catalytic activity of biochar [13,14,15,16,17].

Presently, transition metals, especially iron, have been widely used for enhancing the activation efficiency of biochar due to its relatively low cost and high activity [18,19]. The introduction of iron can not only greatly improve the catalytic performance of biochar, but the recycling process ascribed to the magnetism of Fe^0^ is also fascinating. However, the catalytic performances of pure iron or iron compounds are generally inhibited by solution pH and the accumulation of ferric oxide sludge, thus reducing the number of active sites and ultimately affecting the catalytic performance [20,21]. Fortunately, doping heteroatoms has been proposed and regarded as a feasible way through which to solve this problem [22]. As reported, heteroatom doping could modulate the inherent electroactive sites of a carbon configuration [23]. It not only provides carbocatalysts with a better catalytic efficacy, but it is also expected to trigger non-radical persulfate activation, which exhibits higher selectivity than free radical-based oxidation, thus resulting in a more sufficient degradation toward the target contaminants in complex environments [24]. Numerous studies have found that heteroatom-doped iron-based catalysts have showed a much higher degradation rate toward organic pollutants when compared with only iron-based catalysts [25,26,27]. For example, according to Yu et al.’s study, the N-doped Fe/biochar catalyst showed a 1.7 times higher performance in terms of degrading azo dye acid orange 7 than a Fe/biochar catalyst during the persulfate activation process. As reported in Lv et al.’s research, compared with the Fe@C obtained from MIL-101 (Fe), the S-doped Fe@C exhibited much higher performance (1.5 times larger) in the context of PDS activation.

Boron (B) is another ideal co-doped heteroatom due to its low electronegativity and having an atomic size similar to carbon. Positively charged B doping can easily adjust the electron distribution as well as the physical and chemical properties of sp^2^ hybrid carbon, thus providing more defect sites [28,29]. Moreover, boron doping allows oxygen to be grafted onto the carbon surface, improving the chemical stability of the carbon material [30]. To date, many studies have proven that B doping can significantly improve the activity and stability of catalytic materials [31,32,33]. However, the effect of B doping on an iron-based biochar catalyst has not been clearly investigated, and the theoretical bases still need to be studied in biochar that possess more complicated structures.

Based on the above discussions, fabricating a B and iron co-doped biochar catalyst seems to be a desirable method for PDS activation. Herein, guaiacol (GL), a model compound of lignin, usually exists in pulping wastewater, and it was selected as the target compound due to its inefficient removal by other AOP technologies, such as the Fenton process [34], ozonation [35], and photocatalytic degradation [36]. In this paper, the enhanced activation performance of B on Fe-doped biochar was evaluated. Meanwhile, the reactive species and oxidation pathways were also identified through a comprehensive analysis of radical quenching, electron paramagnetic resonance (EPR) studies, and electrochemical tests. Finally, the reusability of B-Fe/biochar was discussed.

## 2. Results and Discussion

### 2.1. Characterizations of B-Fe/Biochar

**Figure 1:**
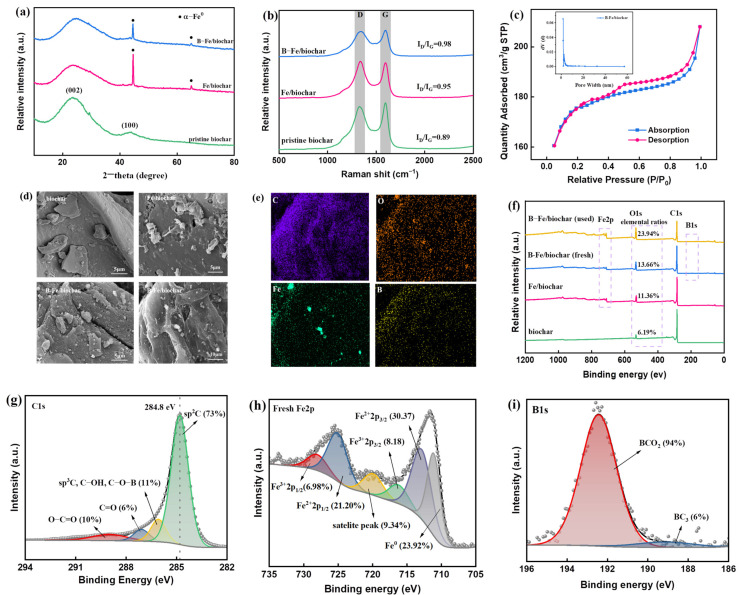
(**a**) XRD patterns; (**b**) Raman spectra. D band corresponds to disordered carbon or defective graphite structure; G band corresponds to E2g mode vibration of complete honeycomb sp2 hybrid carbon grid. (**c**) The nitrogen adsorption–desorption isotherms and pore-size distributions of B-Fe/biochar; (**d**) SEM images; (**e**) SEM mapping images of B-Fe/biochar; (**f**) XPS survey spectra; (**g**) high-resolution XPS spectrum of C 1s; (**h**) high-resolution XPS spectrum of B 1s; (**i**) high-resolution XPS spectrum of Fe 2p.

The phase compositions of pristine biochar, Fe/biochar, and B-Fe/biochar were characterized by XRD patterns. As illustrated in Figure 1a, two diffraction peaks at approximately 26° and 43.6° were associated with the (002) and (100) planes of amorphous and crystalline carbon, respectively [37]. The (002) diffraction peak of B-Fe/biochar was more intense compared to Fe/biochar. The reason for this might be ascribed to the boron-doping process, which might lead to the deformation of the carbon layer and could produce more defects [28]. Meanwhile, both Fe/biochar and B-Fe/biochar presented sharp diffraction peaks at 2θ = 44.5°and 65.0° when assigned to the (100) and (200) planes of α-Fe0 (JCPDS 06-0696), thus demonstrating the successful formation of α-Fe0 in Fe/biochar and B-Fe/biochar [38]. Moreover, no peaks of iron oxide were observed in the XRD patterns for Fe/biochar and B-Fe/biochar, implying that α-Fe0 was the only iron crystal species in the composites. As exhibited in Figure S1, the S-shape curve of B-Fe/biochar reflected its super-paramagnetism with a saturation magnetization (Ms) of 32.0 emu/g, implying that the obtained B-Fe/biochar possess a property of robust magnetism and could be separated from the solution by an external magnet.

The defects and local structural change in samples were further investigated using Raman spectroscopy (Figure 1b). It has been widely recognized that the D peak is associated with the defects of disordered sp^2^-hybridized carbon, while the G peak originates from the crystalline graphite structure in porous carbon [39]. Therefore, the intensity of the ratio of the D peak to the G peak (I_D_/I_G_ ratio) can unveil the defective degree of carbon materials. As illustrated in Figure 1c, the B-Fe/biochar displayed the highest defective level (I_D_/I_G_ of 0.98), which meant that B-Fe/biochar provided the most defective sites. As reported previously, numerous defects sites of carbon lays might be the active sites in catalytic reactions [40]. Therefore, B-Fe/biochar with the highest defect density might show higher catalytic performance.

The N_2_ sorption–desorption curve and the pore size distribution of the B-Fe/biochar are depicted in Figure 1c. It was evident that the material showed a typical type I isotherm with a H4 hysteresis loop, indicating the existence of micropores and mesopores with the majority of the mesopores having a diameter of less than 6 nm (see the insert graph in Figure 1c). The corresponding parameters were gathered in Table S1. As illustrated in Table S1, the pore volume of B-Fe/biochar was similar to that of Fe/biochar, but the average pore width was smaller, resulting a higher specific surface area. The Barrett–Joyner–Halenda (BJH) analysis revealed that the specific surface area of B-Fe/biochar was 538.74 m^2^ g^−1^, which was significantly higher than that of pristine biochar (460.97 m^2^ g^−1^) and Fe/biochar (473.14 m^2^ g^−1^) (Table S1). Thus, B/Fe co-doping allowed the carbon-based catalyst to have a larger specific surface area and more abundant porous structure, thereby promoting the adsorption and transfer of PDS and organic pollutants during the catalytic degradation process.

SEM was conducted to investigate the surface change in biochar during the doping process (Figure 1d). The results displayed that the pristine biochar exhibited an irregular blocky structure with a relatively smooth and flat surface. After Fe doping, irregular pores and cracks were generated on the surface of biochar, which might be caused by the oxidation of K_2_FeO_4_ and corrosion of K (Equations (S1)–(S6)). When further doped with boron, the surface became more rough and uneven, and some pores might be hidden under the fracture surface, which was mainly due to the porogenic effect of boric acid. In addition, many particles were observed on the surface of B-Fe/biochar. Combined with the analysis of XRD patterns, these particles were mainly α-Fe^0^. EDS analysis confirmed that both Fe and B elements were successfully doped into the biochar matrix (Figure 1e).

The surface elements of the prepared catalysts were analyzed by XPS (Figure 1f–i). The spectra in Figure 1f showed that C, O, B, and Fe elements could be detected in the B-Fe/biochar, further confirming the successful doping of B and Fe onto the carbon matrix. Figure 1g depicted that the C 1s spectrum can be decomposed into four components. The main peak centered at 284.8 eV corresponded to the sp^2^ hybridized carbon, indicating the existence of high graphitic carbon in B-Fe/biochar. The tail of the C 1s peak was asymmetric obviously, which implied the disordered structure of graphite carbon. The above results were consistent with those of Raman spectroscopy [41]. The binding energy peaked at 285.8 eV, 287.2 eV, and 289.2 eV, revealing the presence of C–OH/C–O–B, C=O, and O–C=O groups, respectively [28]. The Fe 2p spectrum (Figure 1h) showed five forms of Fe: namely, Fe^0^ (711.1 eV), Fe^2+^ 2p_3/2_ (712.9 eV), Fe^3+^ 2p_3/2_ (716.3 eV), Fe^2+^ 2p_1/2_ (719.9 eV) and Fe^3+^ 2p_1/2_ (728.0 eV) [42]. The presence of Fe (II) and Fe (III) might be attributed to the oxidation of α-Fe0 during storage and analysis. The high-resolution XPS spectrum of B 1s of B-Fe/biochar was also illustrated. As shown in Figure 1i, the binding energies of the two species were 189.2 eV and 192.4 eV, which were attributed to -BC_3_ and -BCO_2_, respectively. The relatively high content of -BCO_2_ was ascribed to the easy bonding of boron atoms to the more electronegative oxygen atoms at the edge sites. The much higher oxygen content of boron-modified biochar compared to boron-free biochar verifies the grafting of oxygen to the carbon surface, resulting in a higher hydrophilicity of the carbon material (Figure 1f).

### 2.2. Catalytic Degradation of GL with B-Fe/Biochar-Activated PDS System

**Figure 2:**
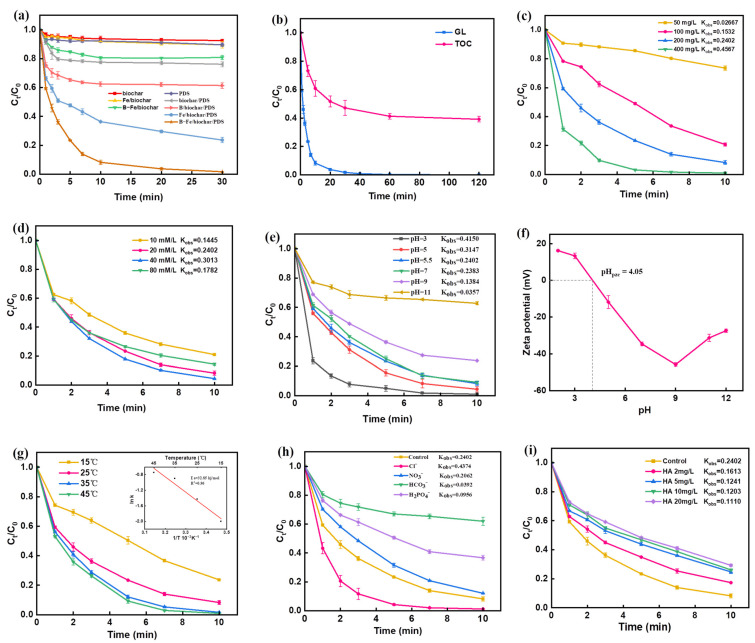
(**a**) GL removal by various catalysts; (**b**) TOC removal in B-Fe/biochar/PDS/GL system; (**c**) effects of B-Fe/biochar dosage; (**d**) effect of PDS dosage; (**e**) effects of initial pH; (**f**) effects of reaction temperature; (**g**) the point of zero charge (pHpzc) of B-Fe/biochar; (**h**) effects of different anions (Cl^−^, NO_3_^−^, HCO_3_^−^, H_2_PO_4_^−^); (**i**) effects of humic acid. Conditions: [Catalyst] = 200 mg/L, [PDS]0 = 20 mM/L, [GL]0 = 50 mg/L, pH ≈ 5.5, T = 25 °C.

The degradation of the GL catalyzed by B-Fe/biochar/PDS system was used to evaluate the activating performances of B-Fe/biochar on PDS. As a control, biochar or Fe/biochar was also tested under identical conditions. As depicted in Figure 2a, GL could not be degraded when only PDS or biochar was present. The slight decrease in concentration of GL might be attributed to the adsorption of biochar. Conversely, in the B-Fe/biochar/PDS system, the removal efficiency of GL within 30 min increased from 19.18% to 98.30%, suggesting the activation of PDS by B-Fe/biochar. Notably, in the B-Fe/biochar/PDS system, the removal rate of GL within 30 min was significantly higher than those of the control group (76.4% for Fe/biochar and 38.6% for biochar). Meanwhile, the total organic carbon (TOC) values of the solutions were also measured, yielding a value of 39.17% (Figure 2b), indicating that more than half of the target pollutant was completely oxidized to H_2_O and CO_2_ by the end of the catalytic reaction (Figure 2b). The mineralization could reach more than 60%. All the results strongly confirmed that the doped B significantly promoted the PDS activation and achieved the rapid degradation of GL.

The effects of important factors, such as catalyst dosage, PDS dosage, pH and co-existing ions, on the GL degradation in the B-Fe/biochar/PDS system were also investigated. Figure 2c illustrated the degradation of GL and corresponding apparent rate coefficients (*K_obs_*) (calculated by Equation (S7)) in the B-Fe/biochar/PDS system within 10 min under various B-Fe/biochar dosages. The results showed that the dosage increased from 50 to 400 mg·L^−1^, while the degradation rate increased from 26.44% to 99.00% with the corresponding *K_obs_* rising from 0.0267 min^−1^ to 0.4567 min^−1^, implying that higher dosages of B-Fe/biochar promoted the degradation of GL. The PDS dosage also played a crucial impact role in GL degradation. The effect of PDS dosage on GL degradation was demonstrated in Figure 2d, which showed that the corresponding *K_obs_* within 10 min rose from 0.1445 to 0.3013 min^−1^ when the PDS dosage increased from 10 to 40 Mm/L. However, with the further increase in PDS concentration, the removal of GL was inhibited, thus leading to a decrease in *K_obs_*. An excess amount of PDS might react with the generated active species, thus reducing the catalytic performance. As shown in Equations (S8) and (S9), the excess SO_4_^−^· produced by PDS could be scavenged by itself to form S_2_O_8_^2−^ or react with S_2_O_8_^2−^. The produced S_2_O_8_^2−^ could further react with ·OH, thus reducing the activity of the system (Equation (S10)) [43,44].

The effect of pH on degradation was investigated because pH played an important role in the degradation process of GL. As shown in Figure 2e, the GL degradation rates and the corresponding *K_obs_* gradually decreased as the pH value increased from 3.0 to 11.0. Compared with the narrow effective pH range of the Fenton reaction (pH = 2.5–4.0), the B-Fe/biochar/PDS system maintained a good degradation performance in a wider pH range of 3.0–9.0, which was favorable for practical applications. The decrease in reaction rate under alkaline conditions could be attributed to the following two reasons [45]. First, the formation of iron hydroxide precipitation was more likely under alkaline conditions, which would hinder the activation reaction of active sites on the catalyst surface with PDS. (2) Secondly, the surface of B-Fe/biochar was negatively charged at pH > 3 (Figure 2f). Additionally, the GL existed as an anion under alkaline conditions (pK_a_ = 9.98), creating electrostatic repulsion between GL and B-Fe/biochar, which was unfavorable for degradation. The effect of temperature on degradation was also studied (Figure 2g). The results showed that the catalytic effect was significantly enhanced with the increase in the reaction temperature, and the GL removal rate increased from 76.39% (15 °C) to 99.10% (45 °C) within 10 min, demonstrating that a higher reaction temperature was beneficial to the electron transfer between the catalyst and PDS. Additionally, a high correlation coefficient (R^2^ = 0.96) between ln k and 1/T was obtained using the pseudo-first-order kinetics. The activation energy (Ea) of the GL degradation system was calculated using the Arrhenius equation and the value was 32.85 kJ/mol, which was lower than that of other catalysts (Table S2) [28,46,47,48,49,50,51], implying that the reaction was easily achieved.

Given the presence of several inorganic anions and humic acid (HA) in the real wastewater, the effects of a variety of inorganic anions (Cl^−^, NO_3_^−^, HCO_3_^−^, H_2_PO_4_^−^) and HA on degradation were evaluated. As demonstrated in Figure 2h, four anions showed certain impact on the degradation of GL. Cl^−^ had a facilitative effect on the degradation of GL. It is speculated that a series of Cl species was generated by the reaction of Cl^−^ with SO_4_^−^· and ·OH, including Cl·, Cl_2_^−^·, Cl_2_ and HClO (Equations (S11)–(S14)), especially the highly reactive HClO counteracted the possible radical scavenging [52,53]. The slight negative influence of NO_3_^−^ in the reaction system might be related to the reason that NO_3_^−^ could react with SO_4_^−^·/OH· and generate free radicals with low redox potential, such as NO_3_· (Equations (S15) and (S16)) [42]. Similarly, the HCO_3_^−^ and H_2_PO_4_^−^ could also quench the SO_4_^−^· and ·OH (Equations (S17)–(S20)) [54,55]. HCO_3_^−^ was able to react with S_2_O_8_^2−^ while H_2_PO_4_^−^ can form a complex with dissolved Fe^2+^ to inhibit the production of SO_4_^−^· (Equation (S21)), further exacerbating the inhibitory effect of GL degradation [56,57].

Humic acid (HA), as a natural organic substance commonly found in water, has been proven to have an effect on the PDS activation process [58]. The effect of HA on GL degradation was shown in Figure 2i. As the HA concentration increased from 2 to 5 mg/L, the degradation rate of GL decreased from 82.65% to 75.24% within 10 min, and further increasing the HA concentration to 20 mg/L barely decreased the degradation rate. The inhibitory effect of HA was mainly attributed to the competitive adsorption of HA and PDS, which hindered the generation of active complexes [59,60]. In addition, HA was rich in electron centers, which could compete with the target pollutant for active radicals [61].

Based on the above results, the B-Fe/biochar/PDS system showed good tolerance to inorganic anions and natural organic matter, demonstrating promising prospects for the removal of pollutants from real wastewater.

### 2.3. Activation Mechanism of Persulfate by the B-Fe/Biochar Catalyst

**Figure 3:**
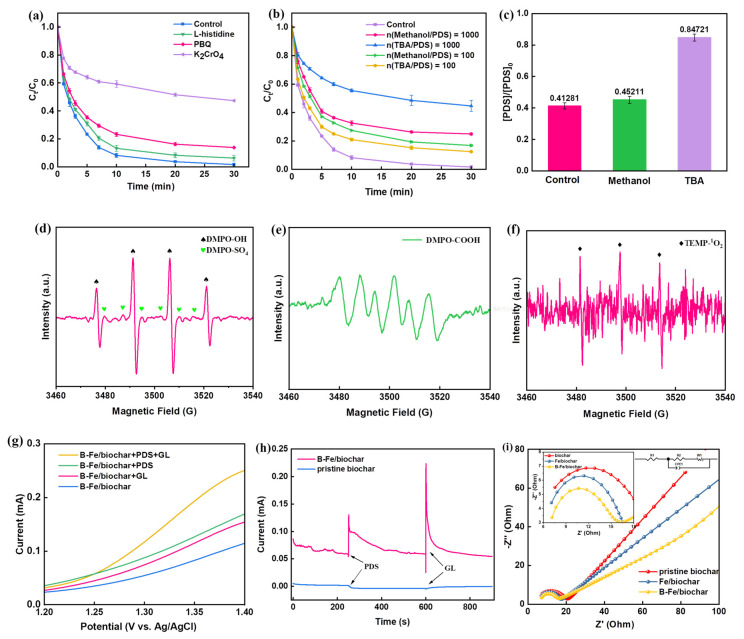
(**a**,**b**) Inhibition of quenching agents and K_2_CrO_4_ on GL degradation; (**c**) PDS decomposition under various quenching agents in the B-Fe/biochar/PDS system; (**d**–**f**) EPR spectra obtained by using DMPO and TEMP as spin-trapping agents; (**g**) linear sweep voltammetry (LSV) curves under different conditions; (**h**) the current-time (i-t) curves of pristine biochar and B-Fe/biochar; (**i**) Nyquist plots of pristine biochar, Fe/biochar and B-Fe/biochar.

It is well known that a variety of reactive oxygen species, such as the radicals like ·OH, SO_4_^−^·, and O_2_^–^· and non-radicals like ^1^O_2_, are generated during the activation of persulfate by Fe-based catalysts [62]. In order to explore the main radicals during the B-Fe/biochar/PDS system, quenching experiments were carried out with different scavengers. Herein, PBQ (k_·O2−_ is 9.6 × 10^8^ M ^−1^·s ^−1^) and L-histidine (k_1O2_ is 2 × 10^9^ M ^−1^·s ^−1^) were chosen as the quenchers for ·O_2_^−^ and ^1^O_2_, respectively. As shown in Figure 3a, the degradation rate of GL decreased by 12.17% after adding PBQ, indicating the presence of ·O_2_^−^ in the reaction system. Meanwhile, the degradation rate of GL decreased slightly when L-histidine was added, which implied that ^1^O_2_ might not be the main active species in the reaction system. Figure 3b presented the degradation of GL after adding methanol and TBA (quenchers for ·OH and SO_4_^−^·). The result showed that both methanol (k_SO4−·_ and k_·OH_ are 1.6–7.8 × 10^6^ M^−1^ s ^−1^ and 1.2–2.8 × 10^9^ M^−1^ s ^−1^, respectively) and TBA (k_SO4−·_ and k_·OH_ are 4–9.1 × 10^5^ M^−1^ s ^−1^ and 3.8–7.6 × 10^8^ M^−1^ s ^−1^, respectively) inhibited the degradation of GL. And the removal efficiencies of GL decreased by 15.13% and 10.80% after the addition of methanol and TBA (n(radical quenchers/PDS) = 100), respectively. The result illustrated that there was both SO_4_^−^· and ·OH in the system. Surprisingly, with the increase in quencher concentration (n (radical quenchers/PDS) = 1000), the inhibition effect of TBA was significantly greater than that of methanol. Generally, it is considered that the inhibition effect of the PDS reaction by methanol is greater than that of TBA due to the higher reaction rate of methanol with SO_4_^−^· and ·OH. The contradictory phenomena observed in this study might be ascribed to the following two reasons. (1) The CH_2_OH· generated from SO_4_^−^· that was scavenged by methanol could react with PDS to produce SO_4_^−^· and then directly participate in the process of GL degradation, thus weakening the inhibitory effect of methanol [63]. (2) TBA had a strong affinity with the surface of carbon, which hindered the contact between PDS and B-Fe/biochar [40]. Figure 3c confirmed the obvious inhibitory effect of TBA on PDS decomposition, thus reducing the reaction rate.

Considering the uncertainty of the quenching results, EPR analysis was further applied to confirm the reactive oxygen species generated in the B-Fe/biochar/PDS system. 5,5-dimethyl-1-pyrrolidone-2-oxyl (DMPO) and 2,2,6,6-tetramethylpiperidine-N-oxyl (TEMP) were chosen as the spin-trapping agents to capture ·OH, SO_4_^−^·, O_2_^−^ and ^1^O_2_, respectively. As shown in Figure 3d, the EPR with DMPO as the trapping agent illustrated weak six signals (1:1:1:1:1:1) and strong four signals (1:2:2:1), suggesting the presence of DMPO-SO_4_ adducts and DMPO-OH adducts, respectively. The result indicated that a large amount of ·OH and a small amount of SO_4_^−^· were produced during the reaction. In addition, the electron paramagnetic resonance spectrum of ·O_2_^−^ captured by DMPO was detected (Figure 3e), meaning the formation of ·O_2_^−^ during the reaction. The role of ·O_2_^−^ was to generate ^1^O_2_ by complexation in the form of superoxide intermediates [64]. However, when TEMP was added, weak triplet signals belonging to TEMP-^1^O_2_ were detected (Figure 3f), further confirming that ^1^O_2_ was probably not the main reactive oxygen species in the reaction system. To confirm whether there was electron transfer between organic matter (electron donor) and persulfate (electron acceptor) [65], K_2_CrO_4_ was added to the PDS system as an electron-trapping agent. As shown in Figure 3a, obvious inhibition was observed, proving the occurrence of electron transfer. When both PDS and GL attached to the B-Fe/biochar, a ternary system with B-Fe/ biochar as the electronic medium was formed, and electron was transferred from adsorbed GL (electron donor) to metastable B-Fe/biochar-PDS complexes (electron receptor). Based on the above discussion, it was deduced that ·OH and direct electron transfer were the main reason for GL degradation in the B-Fe/biochar/PDS system.

The mediated electron transfer mechanism was further confirmed by electrochemical evaluation to clarify the electron transfer in the ternary system. As illustrated in linear sweep voltammetry (LSV) curves (Figure 3g), compared with that when only B-Fe/biochar existed, the current increased after adding PDS, which could be attributed to the interaction of PDS with the catalyst and the formation of metastable reaction complexes on the surface. The current also increased with the addition of GL, verifying the establishment of the electron transfer pathway based on the ternary system. A similar conclusion could be drawn from the i-t plot with the sequential addition of PDS and GL, as shown in Figure 3h, where a significant current jump occurred after the addition of GL. Simultaneously, the property of carbonaceous material also played a key role in the non-radical degradation route, since the current change in pristine biochar was much lower than that of B-Fe/biochar. Moreover, it was concluded from the electrochemical impedance spectrum that the impedance of the carbon-based catalyst was an important parameter that affected the electron transfer. Figure 3i presented the Nyquist plots and the corresponding fitted curves as well as the equivalent circuit model, and it was shown that the semicircle diameter of B-Fe/biochar was smaller than that of Fe/biochar and pristine biochar, indicating that the incorporation of Fe and B effectively reduced the resistance of the biochar, which was beneficial to the fast electron transfer. Thus, B-Fe/biochar had the ability to serve as an electron mediator to induce the electron transfer pathway. The above analysis further confirmed the important role of direct electron transfer in the non-radical pathway during the degradation of GL by the B-Fe/biochar/PDS system.

**Figure 4:**
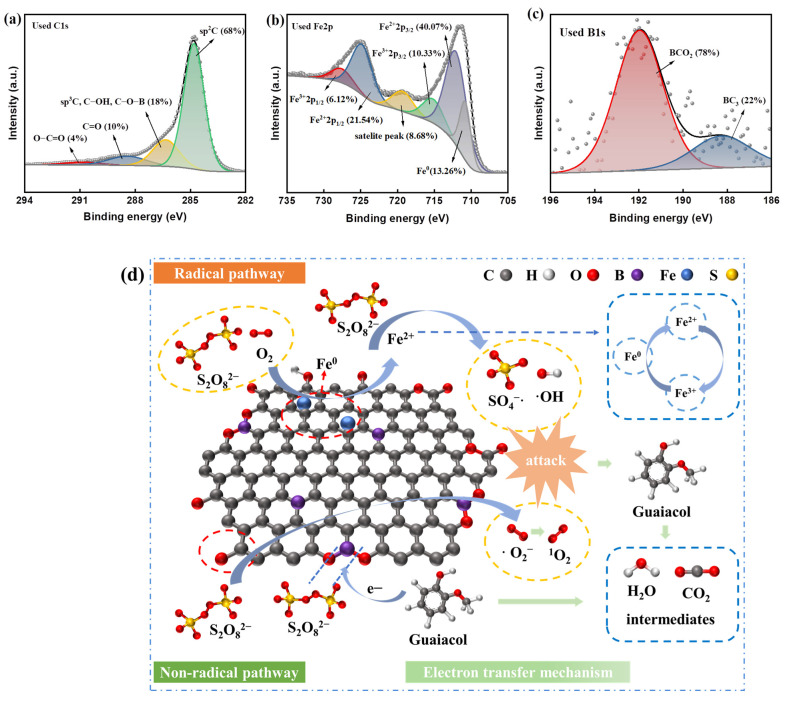
High-resolution XPS spectrum of (**a**) C 1s, (**b**) Fe 2p and (**c**) B 1s of used B-Fe/biochar catalyst; (**d**) mechanism of GL degradation by B-Fe/biochar catalytic activation of PDS.

Additionally, the high-resolution XPS spectra of B-Fe/biochar after activation were also investigated to confirm the activation mechanism. The results are illustrated in Figure 4a–c. It could be seen from Figure 4a that after the activation reaction, the proportion of C=O increased significantly, while the oxygen content also increased significantly. This phenomenon could be attributed to the following two reasons [66]. (1) As a bridge of electron transfer, surface functional groups were oxidized by donating electrons to activate PDS. (2) The high oxidation environment formed by the large number of free radicals generated during the reaction process led to the oxidation of the surface of B-Fe/biochar. The high-resolution XPS spectrum of Fe 2p for used B-Fe/biochar is presented in Figure 4b; compared with the spectrum of fresh B-Fe/biochar (Figure 1h), the amounts of both O and Fe^2+^ increased while Fe^0^ decreased after the reaction, which might be attributable to the increase in iron oxide. Figure 4c showed the high-resolution XPS spectrum of B 1s for used B-Fe/biochar. It was reported that -BCO_2_ as the main activation site could increase the adsorption energy (Eads) between PDS and catalyst and thus enhance the oxidation capacity of the adsorbed PDS [67,68]. From the change in -BCO_2_ content in the catalyst after the reaction (from 94% to 78%) (Figure 4c), it could be deduced that -BCO_2_ was also the catalytic site for the activation of PDS. B doping could promote the interaction between PDS and the catalyst to form metastable surface complexes (Equation (S22)) [69]. Additionally, boron-doped catalysts had lower electrochemical impedance and better electron transfer ability. These metastable complexes were activated in the presence of the contaminant (GL), which acquired electrons from the contaminant, causing the degradation of pollutants and the decomposition of PDS to sulfate ions.

Based on the above results, a possible mechanism of PDS activation by B-Fe/biochar was proposed (Figure 4d). In the free radical pathway, PDS was firstly adsorbed onto the catalyst surface to react with Fe^0^ due to the large specific surface area of B-Fe/biochar. Then, the Fe^0^ could react with O_2_ (Equations (S23) and (S24)) to maintain the continuous release of Fe^2+^, and the resulting Fe^2+^ further activated PDS to produce SO_4_^−·^ and ·OH (Equations (S25) and (S26)). Meanwhile, Fe^0^ reduced part of Fe^3+^ to Fe^2+^ (Equation (S27)), which ensured the concentration of Fe^2+^ in the reaction system and avoided the accumulation of Fe^3+^. The XPS spectra of Fe 2p (Figure 1h and Figure 4b) showed that the proportion of Fe0 decreased from 23.92% to 13.26% after the reaction, while the proportion of Fe^2+^ (from 30.37% to 40.07%) and Fe^3+^ (from 8.18% to 10.33%) increased. These results further verified that α-Fe^0^ formed during the catalyst preparation dominated the activation process of PDS in the free radical pathway. In the non-radical pathway, electron-rich regions on B-Fe/biochar (sp^2^-carbon with defective edges and ketone groups) affected the electronic configuration of PDS (especially the O–O bond in S_2_O_8_^2−^), activating the hydrolysis of PDS, and the resulting HO_2_^−^ would further react with S_2_O_8_^2−^ to form SO_4_^−^· and ·O_2_^−^ (Equations (S28) and (S29)) [70]. Fe^2+^ could also react with O_2_ to form ·O_2_^−^. Under acidic conditions, ·O_2_^−^ complexes could form ^1^O_2_ (Equations (S30) and (S31)) [40]. The direct electron transfer in the non-radical pathway also contributed to the degradation of GL. In general, both free radicals (SO_4_^−^·, ·OH and ·O_2_^−^) and non-free radicals (^1^O_2_ and direct electron transfer) were involved in the degradation of GL, in which ·OH and direct electron transfer played a dominant role.

### 2.4. Stability and Reusability of B-Fe/Biochar Catalyst

**Figure 5:**
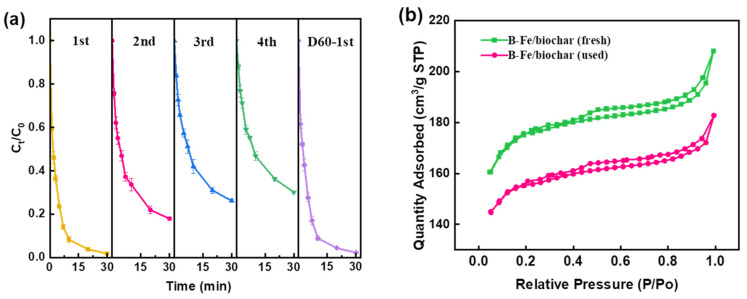
(**a**) Stability and reusability of B-Fe/biochar; (**b**) the nitrogen adsorption–desorption isotherms of fresh and used B-Fe/biochar. Oxidation conditions: [Catalyst] = 200 mg/L, [PDS]_0_ = 20 mM/L, [GL]_0_ = 50 mg/L, pH ≈ 5.5, T = 25 °C.

The stability and reusability of B-Fe/biochar-activated PDS for the degradation of GL were evaluated. As shown in Figure 5a, the degradation performance of B-Fe/biochar stored in air for 60 days still maintained good catalytic performance, while the removal efficiency of GL decreased from 98.30% to 69.93% after three cycles of reuse (Figure 5a). The GL degradation rate decreased with the increase in reuse cycles mainly due to the following two reasons: (1) the inevitable consumption of α-Fe^0^ during the reaction; and (2) the decrease in contact area between B-Fe/biochar and PDS caused by the coverage of GL and degradation products on the biochar [71]. This coincided with the N_2_ adsorption–desorption curves of B-Fe/biochar before and after the catalytic reaction (Figure 5b), and the specific surface area of B-Fe/biochar decreased after the reaction. Further functionalization of the used biochar might improve its performance, but the specific functionalization treatments need to be further explored. In addition, the leaching of Fe during the catalytic reaction was examined. Compared to Fe/biochar (iron leaching 2.21 mg/L), B-Fe/biochar showed much lower iron leaching in the reaction (1.05 mg/L), exhibiting satisfactory long-term stability and reusability. The reason might be ascribed to the smaller pore size of B-Fe/biochar, in which iron particles were not easy to leach.

## 3. Material and Methods

### 3.1. Materials and Chemicals

Masson pine bark was obtained from the forest farm of Zhouning County, Ningde city, Fujian province. The Masson pine bark was washed, dried, pulverized into fine biomass powder and passed through a 100-mesh screen for use. Guaiacol (98.0%), boric acid (99.0%), potassium ferrate (K_2_FeO_4_, 99.0%), and sodium persulfate (Na_2_S_2_O_8_, 99.0%) were purchased from Aladdin Industrial Co., Ltd. (Shanghai, China). Methanol (chromatographically pure) was obtained from Merck Chemical Co., Ltd. (Shanghai, China). Sodium chloride (NaCl, 99.0%), sodium nitrate (NaNO_3_, 99.0%), sodium bicarbonate (NaHCO_3_, 99.0%), sodium dihydrogen phosphate (NaH_2_PO_4_, 99.0%), potassium iodide (KI, 99.0%), tert-butanol (TBA, 99.0%), furfuryl alcohol (FFA, 98%), p-benzoquinone (p-BQ, 99.9%), and potassium chromate (K2CrO4, 99.5%) were purchased from Sinopharm Chemical Reagent Co., Ltd. (Shanghai, China). 5,5-dimethyl-1-pyrroline n-oxide (DMPO, 99.0%) and 2,2,6,6-tetramethyl-1-piperidinyloxy (TEMP, 99.0%) were obtained from Macklin Biochemical Co., Ltd. (Shanghai, China). Deionized water (18.2 MΩ cm^−1^) was used during all experiments.

### 3.2. Synthesis of Boron and Iron Co-Doped Biochar Catalyst (B-Fe/Biochar)

Masson pine bark (5 g, 100 mesh) and boric acid (5 g) were mixed in 50 mL ethanol and stirred at 55 °C for 4 h to evaporate the solvents. After grinding, the dried powder was then transferred into a tube furnace and pyrolyzed at 800 °C under N_2_ for 2 h with a heating rate of 5 °C min^−1^. The obtained product (1 g) was added to 50 mL of K_2_FeO_4_ solution (0.05 M) and then magnetically stirred at room temperature for 12 h. After reaction, the product was filtered, dried, and activated by calcining at 900 °C for 1 h under N_2_ atmosphere. The resulting product was designated as B-Fe/biochar. For comparison, the pristine biochar was prepared via the same procedure without boric acid and K_2_FeO_4_, and Fe/biochar was obtained without boric acid.

### 3.3. Characterization Methods

The morphology of the catalyst was observed by a scanning electron microscope (SEM, Thermo Scientific Verios G4, Thermo Fisher Scientific, Waltham, MA, USA). The microscopic image of biochar was verified by an energy spectrometer (EDS). The nitrogen adsorption–desorption isotherm was measured on an automatic surface analyzer (Micromeritics ASAP 2020 HD88, Micromeritics, Norcross, GA, USA), and the multipoint Brunauer–Emmett–Teller (BET) method and Barrett–Joyner–Halenda (BJH) model were used to calculate the specific surface area and pore size of the catalyst. The magnetic hysteresis curve (M-H curve) was analyzed on a vibrating sample magnetometer (VSM, Lakeshore 7404, Lake Shore Cryotronics, Westerville, OH, USA). The crystalline structures were analyzed by X-ray diffraction (XRD) patterns, which were measured on an X-ray diffractometer (Bruker D8 Advance, Bruker Scientific Technology Co., Saarbrucken, Germany). Raman spectra were investigated on a Renishaw invia Raman microscope (Renishaw InVia reflex, Renishaw Company Ltd, Gloucestershire, UK) with laser excitation at 532 nm. The chemical states of elements were investigated by an X-ray photoelectron spectrometer (XPS, Thermo Scientific K-Alpha, Thermo Fisher Scientific, Waltham, MA, USA). The zeta potential of the catalyst under different pH solutions was determined with a NanoBrook Omni apparatus (NanoBrook Omni, Brookhaven Instruments Corporation, Holtsville, NY, USA). Electron paramagnetic resonance (EPR) spectra were recorded using a Bruker EMXplus-6/1 (Bruker Scientific Technology Co., Saarbrucken, Germany). The electrocatalytic performance of catalysts was tested using a CHI 760E (Shanghai, China). The Fe ion concentration in the reaction solution was determined by inductively coupled plasma mass spectrometry (ICP-MS, Thermo Scientific XSeries 2, Thermo Fisher Scientific, Waltham, MA, USA).

### 3.4. Degradation of GL

The degradation of GL was conducted in a 100 mL triangular flask. First, 20 mg of catalysts and PDS (2 mM) were added into 100 mL of GL solution (50 mg/L). The reaction was then carried out under the speed of 180 rpm at 25 °C The pH of the reaction mixture was approximately 5.5 after PDS addition without adding any buffer. At a certain time interval, 1 mL aliquots of reaction solution were withdrawn and filtered through a 0.22 μm millipore film and then injected into a vial containing 1 mL methanol as a quencher. For the stability test, the catalyst was collected, washed with deionized water and ethanol three times to remove adsorptive pollutants, and then dried at 80 °C for the next run. The GL concentration was analyzed using high-performance liquid chromatography (methanol/water = 40/60, volume ratio) at the wavelength of 274 nm. The concentration of PDS during the reaction was determined using the spectrophotometry method proposed by Liang et al. [72]. Briefly, 100 μL of sample was added into a 10 mL colorimetric tube, which was followed by the successive addition of 4.9 mL solution, which consisted of 0.415 g KI and 0.02 g NaHCO_3_. After reaction for 20 min, the spectrophotometry was performed at a detection wavelength of 352 nm. All the experiments were conducted in triplicate.

GL removal efficiency was determined by Equation (1).

(1) $$Removal\;efficiency\;(\%)=\frac{C_{0}-C_{t}}{C_{0}}\times 100\%$$

where C_t_ is the concentration of GL at time t, mg/L; and C_0_ is the initial concentration of GL, mg/L.

## 4. Conclusions

In summary, we successfully fabricated a B and Fe-doped biochar catalyst (B-Fe/biochar) from Masson pine bark. Compared with pristine biochar or single Fe-doped biochar (Fe/biochar), the catalytic performance of B-Fe/biochar in PDS activation for GL degradation was found to be better, and the degradation rate of GL was influenced by B-Fe/biochar and PDS addition, the solution’s pH value, and reaction temperature. The system could maintain good degradation performance in a wider pH range of 3–9, but it was not effective at a higher pH value. The ·OH was confirmed as the main reactive radical, and the direct electron transfer was found to be primarily responsible for the oxidative degradation of GL. The α-Fe0-mediated radical pathway and -BCO_2_-mediated direct electron transfer pathway together dominated the efficient degradation of GL during PDS activation. After four reuse times, the degradation rate of GL was still up to 69.93% within 30 min, showing great potential application of metal and non-metal co-doped biochar in the treatment of lignin-containing wastewater.

## Data Availability

All the data are available within the manuscript.

## Supplementary Materials

The following supporting information can be downloaded at https://www.mdpi.com/article/10.3390/molecules28227591/s1, Figure S1: Magnetic hysteresis of B-Fe/biochar; Table S1: BET surface area, pore distribution and total pore volume of catalysts; Table S2: Comparison of activation energy of different catalysts for degrading organic.

## References

[1] Vineet K., Pradeep V. (2023). A critical review on environmental risk and toxic hazards of refractory pollutants discharged in chlorolign in waste of pulp and paper mills and their remediation approaches for environmental safety. Environ. Res..

[2] Rasheed T., Bilal M., Nabeel F., Adeel M., Iqbal H.M.N. (2019). Environmentally-related contaminants of high concern: Potential sources and analytical modalities for detection, quantification, and treatment. Environ. Int..

[3] Li Z.P., Liu Q.J., Lin Q.T. (2010). The Application Study Progress of Advanced Treatment Technology of Papermaking Wastewater. Trans. China Pulp Pap..

[4] Wang S., Li X.P., Zhang A.L., Jia C.Y. (2013). Study on the Advanced Treatment of Papermaking Effluent by Membrane Separation Technology. Trans. China Pulp Pap..

[5] Xie L., Liu W.J., Tu Y. (2010). Experimental study on advanced treatment of wastewater from waste paper papermaking by coagulation sedimentation. Chin. J. Environ. Eng..

[6] Deng Y., Zhao R.Z. (2015). Advanced oxidation processes (AOPs) in wastewater treatment. Curr. Pollut. Rep..

[7] Choong Z., Lin K.A., Lisak G., Lim T., Oh W. (2022). Multi-heteroatom-doped carbocatalyst as peroxymonosulfate and peroxydisulfate activator for water purification: A critical review. J. Hazard. Mater..

[8] Zhang Z., Li Z., Bai X., Shi J., Hu M., Chai J., Li K., Jin P. (2023). Photosensitive Dye as an Ideal Peroxymonosulfate Activator for Efficient Self-Degradation: A Novel Idea of Using Waste to Treat Waste. Molecules.

[9] Liu B.Z., Huang B.R., Wang Z.Z., Tang L., Ji C.H., Zhao C. (2023). Homogeneous/heterogeneous metal-catalyzed persulfate oxidation technology for organic pollutants elimination: A review. J. Environ. Chem. Eng..

[10] Gao Y., Wang Q., Ji G.Z., Lin A. (2022). Degradation of antibiotic pollutants by persulfate activated with various carbon materials. Chem. Eng. J..

[11] Chen X., Zhang W.X., Chen Z.L. (2023). Efficient activation of persulfate by metallic sulfide mineral for the efficient removal of pesticides: Performance, radical generation and mechanism. J. Clean. Prod..

[12] Lyu H.H., Lim J.Y., Zhang Q.R., Senadheera S.S., Zhang C.C., Huang Q.L., Ok Y.S. (2024). Conversion of organic solid waste into energy and functional materials using biochar catalyst: Bibliometric analysis, research progress, and directions. Appl. Catal. B Environ..

[13] Zeng S.Y., Li K.Q., Xu X., Zhang J.Y., Xue Y. (2023). Efficiently catalytic degradation of tetracycline via persulfate activation with plant-based biochars: Insight into endogenous mineral self-template effect and pyrolysis catalysis. Chemosphere.

[14] Qin X., Cheng S., Xing B., Qu X., Shi C., Meng W., Zhang C., Xia H. (2023). Preparation of pyrolysis products by catalytic pyrolysis of poplar: Application of biochar in antibiotic wastewater treatment. Chemosphere.

[15] Ho S.H., Chen Y.D., Li R.X., Zhang C.F., Ge Y.M., Cao G.L., Ma M., Duan X.G., Wang S.B., Ren N.Q. (2019). N-doped graphitic biochars from C-phycocyanin extracted spirulina residue for catalytic persulfate activation toward nonradical disinfection and organic oxidation. Water Res..

[16] Renda S., Greco G., González B., Palma V., Manyà J.J. (2021). Wheat-Straw-Derived Activated Biochar as a Renewable Support of Ni-CeO_2_ Catalysts for CO_2_ Methanation. Sustainability..

[17] Dehkhoda A.M., Gyenge E., Ellis N. (2016). A novel method to tailor the porous structure of koh-activated biochar and its application in capacitive deionization and energy storage. Biomass Bioenergy.

[18] Shi Q.K., Deng S., Zheng Y.L., Du Y.L., Li L., Yang S.Z., Zhang G.X., Du L., Wang G.F., Cheng M. (2022). The application of transition metal-modified biochar in sulfate radical based advanced oxidation processes. Environ. Res..

[19] Guo L., Zhao L., Tang Y. (2023). Peroxydisulfate activation using Fe, Co co-doped biochar and synergistic effects on tetracycline degradation. Chem. Eng. J..

[20] Rastogi A., Al-Abed S.R., Dionysiou D. (2009). Effect of inorganic, synthetic and naturally occurring chelating agents on Fe(II) mediated advanced oxidation of chlorophenols. Water Res..

[21] Dong Z.L., Zhang Z.J., Jiang Y., Chu Y.Q., Xu J.Y. (2022). Embedding CsPbBr3 perovskite quantum dots into mesoporous TiO_2_ beads as an S-scheme heterojunction for CO_2_ photoreduction. Chem. Eng. J..

[22] Fan X.F., Yu Y.L., Dong S.J., Liu Y.M., Song C.W., Li Q.B., Wang X.F. (2022). Heteroatoms-doped biochar derived from deciduous resource as persulfate catalysts for efficient degradation of pheno. J. Water Process Eng..

[23] Dou J.B., Cheng J., Lu Z.J., Tian Z.Q., Xu J.M., He Y. (2022). Biochar co-doped with nitrogen and boron switching the free radical based peroxydisulfate activation into the electron-transfer dominated nonradical process. Appl. Catal. B Environ..

[24] Ren W., Xiong L.L., Nie G., Zhang H., Duan X.G., Wang S.B. (2020). Insights into the electrontransfer regime of peroxydisulfate activation on carbon nanotubes: The role of oxygen functional groups. Environ. Sci. Technol..

[25] Li X., Jia Y., Zhou M., Su X., Sun J. (2020). High-efficiency degradation of organic pollutants with Fe, N co-doped biochar catalysts via persulfate activation. J. Hazard. Mater..

[26] Jin Q.Q., Chen Z.L., Chen Q., Yan P.W., Zhao S.X., Shen J.M., Li L., Guo F., Kang J. (2021). Structure activity relationship study of N-doped ligand modified Fe(III)/H_2_O_2_ for degrading organic pollutants. J. Hazard. Mater..

[27] Yu Z.D., Ma J.C., Huang X.Y., Lv Y.C., Liu Y.F., Lin C.X., Dou R.N., Ye X.X., Shi Y.X., Liu M.H. (2022). Insights into enhanced peroxydisulfate activation with S doped Fe@C catalyst for the rapid degradation of organic pollutants. J. Colloid Interface Sci..

[28] Liu B., Guo W., Wang H., Si Q., Zhao Q., Luo H., Ren N. (2020). B-doped graphitic porous biochar with enhanced surface affinity and electron transfer for efficient peroxydisulfate activation. Chem. Eng. J..

[29] Yu J.F., Feng H.P., Tang L., Pang Y., Zeng G.M., Lu Y., Dong H.R., Wang J.J., Liu Y.N., Feng C.Y. (2020). Metal-free carbon materials for persulfate-based advanced oxidation process: Microstructure, property and tailoring. Prog. Mater. Sci..

[30] Agnoli S., Favaro M. (2016). Doping graphene with boron: A review of synthesis methods, physicochemical characterization, and emerging applications. J. Mater. Chem. A.

[31] Wan Y.T., Zhang W., Yu Q., Xiu G.L. (2021). Catalytic degradation of bisphenol A by Fe@B-doped porous carbon. Environ. Pollut. Control..

[32] Pan G., Wei J., Xu M., Li J., Wang L., Li Y., Cui N., Li J., Wang Z. (2023). Insight into boron-doped biochar as efficient metal-free catalyst for peroxymonosulfate activation: Important role of -O-B-O- moieties. J. Hazard. Mater..

[33] Hung C.M., Chen W.C., Huang C.P., Cheng J.W., Dong C.D. (2022). Algae-derived metal-free boron-doped biochar as an efficient bioremediation pretreatment for persistent organic pollutants in marine sediments. J. Clean. Prod..

[34] An J., Zhang G., Zheng R., Wang P. (2016). Removing lignin model pollutants with BiFeO_3_–g-C_3_N_4_ compound as an efficient visible-light-heterogeneous Fenton-like catalyst. J. Environ. Sci..

[35] Chang W., Yan X.F., Liu F., Li L. (2017). Study on Degradation of Guaiacol by Catalytic Ozonation Using AlOOH as Catalyst. Trans. China Pulp Pap..

[36] Xie Z.C., Wang Y., Wang P., Zhang L. (2014). W-Doped TiO_2_ Preparation and Photocatalytic Degradation of Guaiacol. Appl. Mech. Mater..

[37] Zhu S.S., Huang X.C., Ma F., Wang L., Duan X.G. (2018). Catalytic removal of aqueous contaminants on N-doped graphitic biochars: Inherent roles of adsorption and nonradical mechanisms. Environ. Sci. Technol..

[38] Ma D.M., Yang Y., Liu B.F., Xie G.J., Chen C., Ren N.Q., Xing D.F. (2021). Zero-valent iron and biochar composite with high specific surface area via K_2_FeO_4_ fabrication enhances sulfadiazine removal by persulfate activation. Chem. Eng. J..

[39] Li X., Qin Y., Jia Y., Li Y.Y., Zhao Y.X., Pan Y.W., Sun J.H. (2021). Preparation and application of Fe/biochar (Fe-BC) catalysts in wastewater treatment: A review. Chemosphere.

[40] Wang H.Z., Guo W.Q., Liu B.H., Wu Q.L., Luo H.C., Zhao Q., Si Q.S., Sseguya F., Ren N.Q. (2019). Edge-nitrogenated biochar for efficient peroxydisulfate activation: An electron transfer mechanism. Water Res..

[41] Wang S.Y., Xia Y., Tan L., Luo H.P., Liu Y.N., Chen H., Jiang F. (2022). Co and N co-doped hierarchical porous carbon as peroxymonosulfate activator for phenol degradation via nonradical pathway mechanism. Colloids Surf. A Physicochem. Eng. Asp..

[42] Xu L., Fu B.R., Sun Y., Jin P.K., Bai X., Jin X., Shi X. (2020). Degradation of organic pollutants by Fe/N co-doped biochar via peroxymonosulfate activation: Synthesis, performance, mechanism and its potential for practical application. Chem. Eng. J..

[43] Park J.H., Wan J.J., Tafti N., Delaune R.D. (2019). Removal of Eriochrome Black T by sulfate radical generated from Fe-impregnated biochar/persulfate in Fenton-like reaction. J. Ind. Eng. Chem..

[44] Montearudo J.M., El-taliawy H., Duran A., Garo G., Bester K. (2018). Sono-activated persulfate oxidation of diclofenac: Degradation, kinetics, pathway and contribution of the different radicals involved. J. Hazard. Mater..

[45] Huang D.L., Zhang Q., Zhang C., Wang R.Z., Deng R., Luo H., Li T., Li J., Chen S., Liu C.H. (2020). Mn doped magnetic biochar as persulfate activator for the degradation of tetracycline. Chem. Eng. J..

[46] Chen X.L., Li H., Lai L., Zhang Y., Chen Y., Li X., Liu B., Wang H. (2023). Peroxymonosulfate activation using MnFe_2_O_4_ modified biochar for organic pollutants degradation: Performance and mechanisms. Sep. Purif. Technol..

[47] Hussain I., Li M., Zhang Y., Li Y., Huang S., Du X., Liu G., Hayat W., Anwar N. (2017). Insights into the mechanism of persulfate activation with nZVI/BC nanocomposite for the degradation of nonylphenol. Chem. Eng. J..

[48] Liang X., Zhao Y., Guo N., Yang Q. (2021). Heterogeneous activation of peroxymonosulfate by Co_3_O_4_ loaded biochar for efficient degradation of 2,4-dichlorophenoxyacetic acid. Colloids Surf. A Physicochem. Eng. Asp..

[49] Song H., Li Q., Ye Y., Pan F., Zhang D., Xia D. (2021). Degradation of cephalexin by persulfate activated with magnetic loofah biochar: Performance and mechanism. Sep. Purif. Technol..

[50] Xie Y., Hu W., Wang X., Tong W., Li P., Zhou H., Wang Y., Zhang Y. (2020). Molten salt induced nitrogen-doped biochar nanosheets as highly efficient peroxymonosulfate catalyst for organic pollutant degradation. Environ. Pollut..

[51] Zhang Y., Jiang Q., Jiang S.M. (2021). One-step synthesis of biochar supported nZVI composites for highly efficient activating persulfate to oxidatively degrade atrazine. Chem. Eng. J..

[52] Lutze H.V., Kerlin N., Schmidt T.C. (2015). Sulfate radical-based water treatment in presence of chloride: Formation of chlorate, inter-conversion of sulfate radicals into hydroxyl radicals and influence of bicarbonate. Water Res..

[53] Ding C.L., Liu Z., Pan S.Y., Zhao C., Wang Z.W., Gao B.Y., Li Q. (2023). Activation of peroxydisulfate via Fe@sulfur-doped carbon-supported nanocomposite for degradation of norfloxacin: Efficiency and mechanism. Chem. Eng. J..

[54] Wang J., Liao Z.W., Ifthikar JShi L., Chen Z.Q., Chen Z.L. (2017). One-step preparation and application of magnetic sludge-derived biochar on acid orange 7 removal via both adsorption and persulfate based oxidation. RSC Adv..

[55] Ghauch A., Tuqan A.M. (2012). Oxidation of bisoprolol in heated persulfate/H_2_O systems: Kinetics and products. Chem. Eng. J..

[56] Lei Y., Chen C.S., Tu Y.J., Huang Y.H., Zhang H. (2015). Heterogeneous degradation of organic pollutants by persulfate activated by CuO-Fe_3_O_4_: Mechanism, stability, and effects of pH and bicarbonate ions. Environ. Sci. Technol..

[57] Weng C.H., Tao H. (2018). Highly efficient persulfate oxidation process activated with Fe0 aggregate for decolorization of reactive azo dye Remazol Golden Yellow. Arab. J. Chem..

[58] Wu L.B., Lin Q.T., Fu H.Y., Luo H.Y., Zhong Q.F., Li J.Q., Chen Y.J. (2022). Role of sulfide-modified nanoscale zero-valent iron on carbon nanotubes in nonradical activation of peroxydisulfate. J. Hazard. Mater..

[59] Pang K.F., Sun W., Ye F., Yang L.H., Pu M.J., Yang C., Zhang Q.C., Niu J.F. (2022). Sulfur-modified chitosan derived N, S-co-doped carbon as a bifunctional material for adsorption and catalytic degradation sulfamethoxazole by persulfate. J. Hazard. Mater..

[60] Xu K.H., Lin Q.T., Fan X.D., Zheng J.L., Liu Y.X., Ma Y.J., He J. (2023). Enhanced degradation of sulfamethoxazole by activation of peroxodisulfate with red mud modified biochar: Synergistic effect between adsorption and nonradical activation. Chem. Eng. J..

[61] Guan C., Jiang J., Pang S., Luo C., Ma J., Zhou Y., Yang Y. (2017). Oxidation kinetics of bromophenols by nonradical activation of peroxydisulfate in the presence of carbon nanotube and formation of brominated polymeric products. Environ. Sci. Technol..

[62] Li X., Zhang S.X., Yu M.W., Xu H., Lv J.G., Yang S.S., Zhu X.T., Li L. (2021). One-pot pyrolysis method for synthesis of Fe/N co-doped biochar as an effective peroxymonosulfate activator for RhB degradation. J. Taiwan Inst. Chem. Eng..

[63] Yu J.F., Tang L., Pang Y., Zeng G.M., Wang J.J., Deng Y.C., Liu Y.N., Feng H.P., Chen S., Ren X.Y. (2019). Magnetic nitrogen-doped sludge-derived biochar catalysts for persulfate activation: Internal electron transfer mechanism. Chem. Eng. J..

[64] Hayyan M., Hashim M.A., Alnashef I.M. (2016). Superoxide ion: Generation and chemical implications. Chem. Rev..

[65] Lee H., Lee H.J., Jeong J., Lee J., Park N., Lee C. (2015). Activation of persulfates by carbon nanotubes: Oxidation of organic compounds by nonradical mechanism. Chem. Eng. J..

[66] Tang L., Liu Y.I., Wang J.J. (2018). Enhanced activation process of persulfate by mesoporous carbon for degradation of aqueous organic pollutants: Electron transfer mechanism. Appl. Catal. B-Environ..

[67] Chen C., Zhou L.L., Huang Y.N., Wang W.K., Xu J. (2022). Boron regulates catalytic sites of biochar to enhance the formation of surface-confined complex for improved peroxydisulfate activation. Chemosphere.

[68] Nie C.Y., Dai Z.H., Liu W.J., Duan X.G., Wang C.Y., Lai B., Ao Z.M., Wang S.B., An T.C. (2020). Criteria of active sites in nonradical persulfate activation process from integrated experimental and theoretical investigations: Boron-nitrogen-co-doped nanocarbon-mediated peroxydisulfate activation as an example. Environ. Sci. Nano.

[69] Ahn Y.Y., Bae H., Kim H.I., Kim S.H., Kim J.H., Lee S.G., Lee J. (2019). Surface-loaded metal nanoparticles for peroxymonosulfate activation: Efficiency and mechanism reconnaissance. Appl. Catal. B-Environ..

[70] Ren X.Y., Wang J.J., Yu J.F., Song B., Feng H.P., Shen M.C., Zhang H., Zou J.J., Zeng G.M., Tang L. (2021). Waste valorization: Transforming the fishbone biowaste into biochar as an efficient persulfate catalyst for degradation of organic pollutant. J. Clean. Prod..

[71] Chen C., Ma T.F., Shang Y.N., Gao B.Y., Jin B., Dan H.B., Li Q., Yue Q.Y., Li Y.W., Wang Y. (2019). In-situ pyrolysis of Enteromorpha as carbocatalyst for catalytic removal of organic contaminants: Considering the intrinsic N/Fe in Enteromorpha and non-radical reaction. Appl. Catal. B-Environ..

[72] Liang C.J., Huang C.F., Mohanty N., Kurakalva R.M. (2008). A rapid spectrophotometric determination of persulfate anion in ISCO. Chemosphere.

