# Supplementary material for: Insights into Enhanced Peroxydisulfate Activation with B and Fe Co-Doped Biochar from Bark for the Rapid Degradation of Guaiacol
Source: Molecules. 2023 Nov 14;28(22):7591. doi: 10.3390/molecules28227591 (PMC10674898; doi:10.3390/molecules28227591)
Supplement: Supplementary file 1 [file molecules-28-07591-s001.zip › molecules-2684056-supplementary.pdf]

Insights into Enhanced Peroxydisulfate Activation with B and Fe Co-Doped Biochar from Bark for the Rapid Degradation of Guaiacol

Jian Huang [1], Yu Zhu [1], Huiyang Bian [2], Liang Song [1], Yifan Liu [1], Yuancai Lv [1], Xiaoxia Ye [1], Chunxiang Lin [1,*] and Xiaojuan Li [1,*]

[1] College of Environment & Safety Engineering, Fuzhou University, Fuzhou 350108, China; hj@fzu.edu.cn (J.H.); zy12500947@outlook.com (Y.Z.); t23087@fzu.edu.cn (L.S.); yfanym@fzu.edu.cn (Y.L.); yclv@fzu.edu.cn (Y.L.); yexiaoxia@fzu.edu.cn (X.Y.)

[2] Jiangsu Co-Innovation Center of Efficient Processing and Utilization of Forest Resources, Nanjing Forestry University, Nanjing 210037, China; hybian1992@njfu.edu.cn

* Correspondence: lcx2010@fzu.edu.cn (C.L.); lixiaojuan@fzu.edu.cn (X.L.); Tel.: +86-0591-2286-6072 (C.L.)

$$4K_2FeO_4 + 10H_2O \rightarrow 4Fe(OH)_3 + 3O_2\uparrow + 8KOH \quad (S1)$$

$$2KOH + CO_2 \rightarrow K_2CO_3 + H_2O \quad (S2)$$

$$K_2CO_3 \rightarrow K_2O + CO_2\uparrow \quad (S3)$$

$$2KOH + 2C \rightarrow 2K\uparrow + H_2\uparrow + 2CO\uparrow \quad (S4)$$

$$K_2CO_3 + 2C \rightarrow 2K\uparrow + 3CO\uparrow \quad (S5)$$

$$K_2O + C \rightarrow 2K\uparrow + CO\uparrow \quad (S6)$$

$$\ln\frac{C_0}{C_t} = K_{obs}t \quad (S7)$$

where C_0 and C_t (mg/L) represent for the concentrations of GL at initial and t (min) time, respectively.

$$SO_4^-\cdot + SO_4^-\cdot \rightarrow S_2O_8^{2-} \quad (S8)$$

$$SO_4^-\cdot + S_2O_8^{2-} \rightarrow SO_4^{2-} + S_2O_8^-\cdot \quad (S9)$$

$$S_2O_8^{2-} + \cdot OH \rightarrow S_2O_8^-\cdot + OH^- \quad (S10)$$

$$Cl^- + SO_4^-\cdot \leftrightarrow Cl\cdot + SO_4^{2-} \quad (S11)$$

$$Cl\cdot + Cl^- \leftrightarrow Cl_2^-\cdot \quad (S12)$$

$$2\,Cl_2^-\cdot \rightarrow Cl_2 + 2\,Cl^- \quad (S13)$$

$$Cl_2^-\cdot + \cdot OH \rightarrow HClO + Cl^- \quad (S14)$$

$$NO_3^- + SO_4^-\cdot \rightarrow NO_3\cdot + SO_4^{2-} \quad (S15)$$

$$NO_3^- + \cdot OH \rightarrow NO_3\cdot + OH^- \quad (S16)$$

$$HCO_3^- + SO_4^-\cdot \rightarrow CO_3^-\cdot + SO_4^{2-} + H^+ \quad (S17)$$

$$HCO_3^- + \cdot OH \rightarrow CO_3^-\cdot + H_2O \quad (S18)$$

$$H_2PO_4^- + SO_4^-\cdot \rightarrow H_2PO_4\cdot + SO_4^{2-} \quad (S19)$$

$$H_2PO_4^- + \cdot OH \rightarrow H_2PO_4\cdot + OH^- \quad (S20)$$

$$S_2O_8^{2-} + HCO_3^- \rightarrow HCO_4^- + SO_4^- + SO_3^- \quad (S21)$$

$$B-Fe/biochar + PDS \rightarrow [B-Fe/biochar-PDS]complex \quad (S22)$$

$$2Fe^0 + O_2 + 2H_2O \rightarrow 2Fe^{2+} + 4OH^- \tag{S23}$$

$$Fe^0 + S_2O_8^{2-} \rightarrow Fe^{2+} + 2SO_4^{2-} \tag{S24}$$

$$Fe^{2+} + S_2O_8^{2-} \rightarrow SO_4^- \cdot + SO_4^{2-} + Fe^{3+} \tag{S25}$$

$$SO_4^- \cdot + H_2O \rightarrow SO_4^{2-} + \cdot OH + H^+ \tag{S26}$$

$$Fe^0 + 2Fe^{3+} \rightarrow 3Fe^{2+} \tag{S27}$$

$$S_2O_8^{2-} + 2H_2O \rightarrow HO_2^- + 2SO_4^{2-} + 3H^+ \tag{S28}$$

$$S_2O_8^{2-} + HO_2^- \rightarrow SO_4^- \cdot + SO_4^{2-} + H^+ + \cdot O_2^- \tag{S29}$$

$$Fe^{2+} + O_2 \rightarrow \cdot O_2^- + Fe^{3+} \tag{S30}$$

$$\cdot O_2^- + 2H^+ \rightarrow {}^1O_2 + H_2O_2 \tag{S31}$$

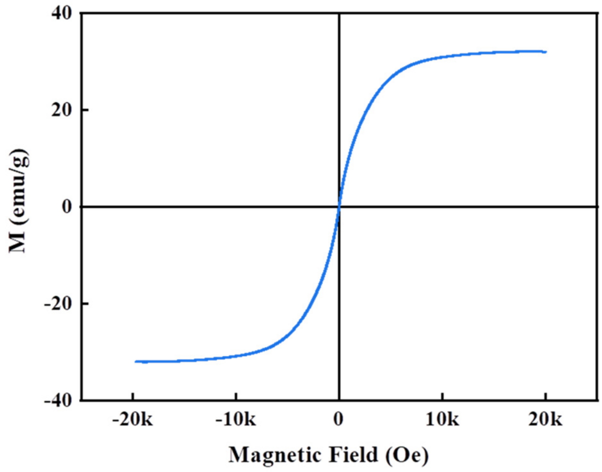

Fig. S1 Magnetic hysteresis of B-Fe/biochar

Table S1. BET surface area, pore distribution and total pore volume of catalysts.

**Catalyst types**	**Specific surface area (m^2 g^{-1})**	**Adsorption average pore width (nm)**	**Pore volume (cm^3 g^{-1})**
Pristine biochar	460.97	2.21	0.25
Fe/biochar	473.14	2.83	0.33
B-Fe/biochar	538.74	2.39	0.32

Table S2 Comparison of activation energy of different catalysts for degrading organic

**Catalyst**	**Target pollutant**	**Activation energy (KJ/mol)**	**Reference**
B-KBC	sulfamethoxazole	63.75	Liu et al, 2020
Fe2O3@LBC	cephalexin	38.76	Song et al, 2021
MnFe2O4/BC	malachite green	84.41	Chen et al, 2023
nZVI@BC	atrazine	83.34	Zhang et al, 2021
CBC-1	2,4-dichlorophenoxyacetic acid	43.73	Liang et al, 2021
NCS-6	bisphenol A	34.1	Xie et al, 2020
nZVI/BC3	nonylphenol	43.45	Hussain et al, 2017
B-Fe/biochar	guaiacol	32.85	This work
